# Post-traumatic stress disorders, anxiety, and depression in college students during the COVID-19 pandemic: a cross-sectional study

**DOI:** 10.1186/s12888-023-04660-9

**Published:** 2023-04-04

**Authors:** Lijuan Quan, Wei Lu, Rui Zhen, Xiao Zhou

**Affiliations:** 1 School of Educational Science, Anhui Normal University, Wuhu, 241000 China; 2 Jing Hengyi School of Education, Hangzhou Normal University, Hangzhou, 311121 China; 3 Department of Psychology and Behavioral Sciences, Zhejiang University, Hangzhou, 310028 China

**Keywords:** COVID-19, College students, PTSD, Depression, Anxiety

## Abstract

**Objectives:**

The COVID-19 pandemic has caused an increase in psychiatric disorders in college students, particularly posttraumatic stress disorders (PTSD), depression, and anxiety. While existing studies assess the prevalence of these disorders and their predictors, they overlook potential complications caused by comorbidity between these disorders. To fill this gap, this study examined the prevalence of PTSD, depression, anxiety, and their comorbidity to inform targeted intervention for college students during the COVID-19 pandemic.

**Design:**

Self-report questionnaires were used to assess 6,898 college students about six months after the COVID-19 outbreak.

**Results:**

The results found that the prevalence of PTSD, depression, and anxiety were 15.5%, 32.2%, and 32.1% respectively, and the prevalence of comorbid PTSD and depression, comorbid PTSD and anxiety, comorbid depression and anxiety, and comorbid PTSD, depression, and anxiety symptoms were 11.5%, 11.6%, 20.4%, and 9.4% respectively. Moreover, left-behind status, lower economic status, previous trauma experiences, exposure to the pandemic, and rumination were risk factors of psychological distress, but self-disclosure was a protective factor for these disorders.

**Conclusion:**

These results indicate that distinct psychiatric disorders may be comorbid in individuals, and are further influenced by pre-, within-, and post-disaster factors. Furthermore, psychological service targeted at college students should pay attention to comorbid symptoms rather than only symptoms of single disorders.

## Introduction

The COVID-19 pandemic posed an existential threat to peoples’ lives and many individuals throughout the world have felt threatened and afraid [1, 2]. In light of these facts, the COVID-19 pandemic was considered a major traumatic event [3], and thus may lead to various negative psychological outcomes [4–6]. Studies found that posttraumatic stress symptoms, depression, and anxiety were relatively common in people during the COVID-19 pandemic [7–11]. For example, a study of 2,205 Chinese college students found that the prevalence of COVID-19-related PTSD was 34.2% [12]. Yu investigated 23,863 Chinese college students and found that 31.9% of them showed mild to severe depression during this pandemic [13]. In Abubakar et al.’s study, 50.5% of Indonesian college students showed anxiety during the COVID-19 pandemic [14]. A study conducted among university students from Germany, Poland, Russia, Slovenia, Turkey, and Ukraine revealed coronavirus-related PTSD risk at three cutoff scores (25, 44, and 50) was 78.20%, 32.70%, and 23.10% [15]. Further research conducted in France, Ethiopia and Malaysia also shown a significant negative impact on the mental health of college students during the COVID-19 pandemic [16–19]. Thus, most researchers concluded that the pandemic led to a 27.6% increase in cases of major depressive disorders and 25.6% increase in cases of anxiety disorders globally [20].

The high prevalence of these disorders raises the question of what factors cause these disorders. Freedy et al.’s theoretical model provided a potential explanation; posttraumatic reactions may be affected by pre-, within-, and post-disaster factors [21]. Here, gender, age, family economic status, previous trauma experiences and other demographic information were considered pre-disaster factors, and suggested that females, young people, and people of low economic status were at higher risk of developing disorders including PTSD, depression, and anxiety [6, 22–24]. Within-disaster factors mainly refer to the exposure to disasters [21], which always have a dose-effect relationship to posttraumatic distress [25]. In light of this, studies found that higher severity of exposure to the COVID-19 pandemic was related to more PTSD, anxiety, and depression [26–29].

A meta-analysis found that compared with the pre-and within-disaster factors, post-disaster factors play a more important role in psychiatric outcomes [30]. Rumination as a type of perseverative cognition which involves repeated and unproductive dwelling on a particular theme [31], is an important post-disaster factor in predicting negative outcomes [32]. It is theorized that individuals with rumination over-focus on the negative aspects of traumatic events, limiting their ability to notice trauma-related cues in their behavior [33]. This, in turn, causes an increase in negative feelings or a depressive state [31, 32], and finally leading to psychiatric disorders such as PTSD, depression, and anxiety. Another post-disaster factor is self-disclosure, which may relieve psychological distress [34, 35]. On one hand, by talking with others, people may clarify their psychological states and come to term with events, which in turn could lead them to reframe the meaning of trauma [36]. On the other hand, disclosure may cause people to habituate gradually to stressful events and thus relieve their negative reactions following these events [37]. This reframing and habituation may mitigate the severity of PTSD, depression, and anxiety. Given these findings, it was proposed that rumination may have exacerbated, and self-disclosure may have relieved, individuals’ PTSD, depression, and anxiety during the COVID-19 pandemic.

While pre-, within-, and post-disaster factors may play a role in psychological distress following trauma, it remains unclear which aspect plays the most important role [32]. Furthermore, existing studies found that during the COVID-19 pandemic, people exhibited many types of negative outcomes, and these symptoms may have been comorbid in these individuals [24]. Therefore, Understanding the impact of the COVID-19 pandemic on people’s mental health and timely screening for related symptoms such as anxiety, depression, PTSD and their comorbidities can help alleviate negative outcomes and psychological treatment responses. Moreover, although the comorbidities between PTSD and depression, and anxiety and depression are well known [38], their triple comorbidity among Chinese college students during pandemic, has not been clarified, limiting the comprehensive understanding of posttraumatic symptoms during this pandemic and thereby reducing the effectiveness of psychological services. To fill these gaps, the aim of this study is to assess the prevalence of PTSD, depression, anxiety, and their comorbidity, followed by examining the predictors for them from the pre-, within-, and post-disaster perspective according to Freedy ’s theoretical model [21].

## Methods

### Participants and procedures

This study began on June 1, 2020, after the COVID-19 outbreak. In the current study, we focused on university undergraduates. Because many universities are still closed in order to reduce the likelihood of teachers and students becoming infected with COVID-19, we conducted an Internet-based survey to assess psychological responses among college students. Using the WeChat platform (a free messaging and calling app used commonly in China), we sent the internet-based questionnaire to college counselors and asked them to send the questionnaires to their students to answer. Using this method, we gathered 6,898 participants in several provinces in China. This project was approved by the Research Ethics Committee of Psychology and Behavioral Sciences, Zhejiang University. Informed consent was obtained from each participant. No compensation was provided to students, and graduate students were excluded from this study.

### Measures

All instruments used in this study were self-report questionnaires. We first collected participants’ demographic information (e.g., gender, grade, single child or not, family monthly income, and previous trauma experiences). Next, we assessed students’ levels of PTSD, depression, anxiety, pandemic exposure, rumination, and self-disclosure.

A Chinese version of the PTSD Checklist for DSM-5 (Weathers, 2013) was used to assess college students’ levels of PTSD [39]. This measure is a 20-item self-report scale designed to assess the occurrence and frequency of PTSD symptoms in relation to the traumatic experienced by an individual. In the present study, all respondents rated the frequency of COVID-19 related symptoms during the previous 2 weeks on a 5-point scale that ranged from 0 (not at all/only once) to 4 (almost every day). The scale has four subscales: intrusions, negative cognition and emotion alteration, avoidance, and hyperarousal. In this sample, the scale demonstrated good internal consistency (Cronbach’s alpha = 0.96) and good structural validity (CFI = 0.89). According to Weathers ’s suggestion, the cutoff score for PTSD is 34 [40].

Depression was assessed by the depression subscale of the Symptom Checklist-90 (SCL-90) [41]. SCL-90 was developed by Dr. Leonard Derogatis [42]. The Chinese version of SCL-90 was introduced and developed by Wang Zhengyu in 1984 and was confirmed to have good reliability and validity by some Chinese scholars [41, 43, 44]. Confirmatory factor analysis indicated a good fit (CFI = 0.90). This subscale has 13 items, which are rated by a 5-point scale ranged 0 (completely disagree) to 4 (completely agree). In this sample, the scale demonstrated good internal consistency (Cronbach’s alpha = 0.90). The cutoff score for depression in this subscale is 13.

Generalized Anxiety Disorder-7 (GAD-7) was used to assess the college students’ anxiety levels [45]. The GAD-7 consists of a self-report questionnaire that allows for the rapid detection of GAD. College students were asked if they were bothered by anxiety-related problems over the past two weeks by answering seven items on a 4-point scale that ranged from 0 (not at all) to 3 (nearly every day). The total score ranges from 0 to 21. The internal consistency of the Chinese version of the GAD-7 has been found to be high. In this sample, the scale demonstrated good internal consistency (Cronbach’s alpha = 0.96) and good structural validity (CFI = 0.96). The cutoff score for GAD-7 is 9.

Exposure to the COVID-19 pandemic was assessed by Zhen and Zhou’s Pandemic Experiences Scale [46]. This scale has ten items (e.g., I was infected during the COVID-19 outbreak, I was quarantined during the COVID-19 outbreak), each item was rated based on “No” and “Yes”, wherein 0 represented “No” and 1 represented “Yes”.

Rumination was assessed by the rumination subscale of the Cognitive Emotion Regulation Questionnaire [47], and confirmatory factor analysis shows a good fit (CFI = 0.99). This subscale has 4 items. All of these items were rated on a 5-point Likert scale, which ranged from 1 (completely disagree) to 5 (completely agree). The current study revealed good internal reliability for this subscale (Cronbach’s alpha = 0.88).

Self-disclosure was assessed by Zhen’s Chinese version of Distress Disclosure Index and confirmatory factor analysis shows a good fit (CFI = 0.90) [36]. The original scale was developed by Kahn and Hessling and then revised by Zhen for Chinese samples. The scale comprises 12 items rated on a 5-point Likert-type scale from 1 to 5 (1 = completely disagree and 5 = completely agree). For this study, the internal reliability of the questionnaire was good (Cronbach’s alpha = 0.78).

### Data analysis procedures

Statistical analyses were performed using SPSS version 20.0. Chi-square tests were used to assess differences in the prevalence of PTSD, depression, anxiety, and their comorbid symptoms among undergraduates by demographic characteristics. Multivariate logistic regression analyses were then performed to examine the role of demographic characteristics, pandemic exposure, rumination, and self-disclosure in PTSD, depression, anxiety, and their comorbid symptoms. Odds ratios (ORs) and 95% confidence intervals (CIs) were used to show the predictive utility of these factors.

## Results

### Sample characteristics

The sample characteristics were in Table 1. Of the 6,898 college students, 1,856 (26.9%) were male students and 5,042 (73.1%) were female students. 3,152 (45.7%), 1,663 (24.1%), 1,688 (24.5%), and 395 (5.7%) college students were in their 1st, 2nd, 3rd, and 4th year of University, respectively. There were 2,563 (37.2%) left-behind students, and 4,335(61.5%) were non-left-behind students. 2,535 (36.7%) students lived in urban areas, and 4,363 (63.3%) students lived in rural areas. 2,657 (38.5%) students were only children, and 4,241 (61.5%) students have siblings. 2,454 (35.6%) students’ family monthly income is lower than 5,000 RMB, and 4,444 (64.4%) students’ family monthly income is more than 5,000 RMB. There were 2,539 (36.8%) exposed to at least one traumatic event in their previous experiences, and 4,359 (63.2%) responded that they had not experienced previous trauma.

**Table 1:** Prevalence of PTSD, depression, and anxiety

Variables	PTSD		Depression		Anxiety		Comorbid PTSD and depression		Comorbid PTSD and anxiety		Comorbid depression and anxiety		Comorbid PTSD, depression, and anxiety	
	%	χ^2^	%	χ^2^	%	χ^2^	%	χ^2^	%	χ^2^	%	χ^2^	%	χ^2^
**Gender**		7.27**		15.55***		0.28		4.20*		1.25		3.41		0.84
Male (n = 1856, 26.9%)	17.4		35.9		32.6		12.8		12.3		22.0		10.0	
Female (n = 5042, 73.1%)	14.8		30.9		31.9		11.1		11.4		20.0		9.2	
**Grade**		11.17*		13.99**		15.36**		15.0**		11.29*		13.4**		12.52**
Grade1 (n = 3152, 45.7%)	15.7		32.5		30.4		11.1		11.6		20.0		9.3	
Grade 2 (n = 1663, 24.1%)	15.7		32.7		33.0		12.1		11.8		20.5		9.5	
Grade 3 (n = 1688, 24.5%)	16.2		33.3		35.3		13.0		12.6		22.9		10.8	
Grade 4 (n = 395, 5.7%)	9.6		23.8		28.4		6.3		6.6		15.2		5.1	
**Left-behind experiences**		4.15*		13.55***		27.35		6.48*		8.28**		19.24***		8.46**
Yes (n = 2563, 37.2%)	16.6		34.9		35.9		12.8		13.1		23.3		10.8	
Non (n = 4335, 61.5%)	14.8		30.6		29.9		10.8		10.8		18.9		8.7	
**Household residence**		0.92		2.07		8.04**		0.38		0.24		0.76		0.56
City (n = 2535, 36.7%)	16.0		31.2		30.0		11.8		11.9		20.0		9.8	
Village (n = 4363, 63.3%)	15.2		32.8		33.3		11.3		11.5		20.9		9.2	
**Only child or not**		0.30		0.94		10.51**		1.21		2.13		4.38*		2.52
Yes (n = 2657, 38.5%)	15.2		31.5		29.2		11.0		10.9		19.3		8.7	
No (n = 4241, 61.5%)	15.7		32.7		33.6		11.9		12.1		21.4		9.9	
**Family month income**		6.07*		9.80**		8.46**		10.52**		8.74**		11.10**		12.08**
≤ 5000 (n = 2454, 35.6%)	16.9		34.6		34.3		13.2		13.2		22.7		11.1	
> 5000 (n = 4444, 64.4%)	14.7		30.9		30.9		10.6		10.8		19.4		8.5	
**Previous trauma experiences**		0.50		19.55***		52.65***		3.94*		9.13**		47.08***		12.49***
Yes (n = 2539, 36.8%)	15.9		35.5		37.5		12.5		13.2		24.9		11.1	
No (n = 4359, 63.2%)	15.2		30.3		29.0		10.9		10.7		18.0		8.5	

Note: * p < 0.05, **p < 0.01,***p < 0.001

### Prevalence of PTSD, depression, and anxiety

The mean values of PTSD, depression, and anxiety are 19.48 (SD = 13.17, range:0 ~ 60), 9.85 (SD = 10.88, range: 0 ~ 52), and 3.34 (SD = 4.38, range: 0 ~ 21). Based on the cutoff scores of 34, 13, and 5 for PTSD, depression, and anxiety respectively, there were 1,067 (15.5%), 2,223 (32.2%), and 2,215 (32.1%) probable PTSD, depression, and anxiety cases, respectively.

Next, we carried out correlation analysis of PTSD, depression, and anxiety. We found that PTSD was associated positively with depression (r = 0.45, p < 0.001;95%CI:0.42 ~ 0.47) and anxiety (r = 0.50, p < 0.001; 95%CI: 0.48 ~ 0.52), and depression showed a positive relationship to anxiety (r = 0.56, p < 0.001; 95%CI: 0.54 ~ 0.59). We then assessed for comorbidity and found that 795 (11.5%) students had comorbid PTSD and depression, 802 (11.6%) students had comorbid PTSD and anxiety, 1,418 (20.4%) had comorbid depression and anxiety, and 651 (9.4%) had comorbid PTSD, depression, and anxiety.

Table 1 shows the results of chi-square tests for the univariate associations between participants’ demographic characteristics and PTSD, depression, anxiety, and their comorbid symptoms. Male students had higher instances of PTSD, depression, and comorbid PTSD and depression symptoms than that of female students. Grade 4 students had lower instances of PTSD, depression, anxiety, and their comorbid symptoms than that of students in other grades. Students who were left-behind, whose family monthly income is lower than 5,000 RMB, and had experienced previous traumatic events, had more prevalence of PTSD, depression, anxiety, and comorbid symptoms. Students from rural areas reported more anxiety than the students from urban areas, and students with siblings had higher instances of anxiety and comorbid depression and anxiety than that of the only children.

### Predictors of PTSD, depression, and anxiety

Multivariate logistic regression analyses were performed to examine associations between demographic characteristics, pandemic exposure, rumination, and self-disclosure and PTSD, depression, anxiety, and their comorbid symptoms. We found that except for gender, all variables were correlated with these symptoms (see Table 2). The results suggested that the 4th year students were less likely to report PTSD, depression, anxiety, and their comorbid symptoms. Compared with non-left-behind students and students who had not experienced previou trauma, left-behind students and students who had previous traumatic experiences were more likely to have depression, anxiety, and their comorbid symptoms. Compared with the students with siblings, the students who are only children were less likely to have anxiety, comorbid depression and anxiety, comorbid PTSD, depression, and anxiety. Family monthly income had a non-significant relationship to anxiety symptoms, but had a significant association with other symptoms. The students whose family monthly income was lower than 5,000 RMB were more likely to have PTSD, depression, comorbid PTSD and depression, comorbid PTSD and anxiety, comorbid anxiety and depression, comorbid PTSD, depression, and anxiety. Furthermore, we also found that rumination was positively correlated correlated with PTSD, depression, anxiety, and their comorbid symptoms, but self-disclosure was negatively correlated. Pandemic exposure was positively correlated with PTSD, anxiety, and their comorbid symptoms.

**Table 2:** Predictors of PTSD, depression, and anxiety

Variables	PTSD ^a^	Depression ^*b*^	Anxiety ^*c*^	Comorbid PTSD and depression ^*d*^	Comorbid PTSD and anxiety ^*e*^	Comorbid depression and anxiety ^*f*^	Comorbid PTSD, depression, and anxiety ^g^
	OR(95%CI)	OR(95%CI)	OR(95%CI)	OR(95%CI)	OR(95%CI)	OR(95%CI)	OR(95%CI)
Male(ref = Female)	0.99 (0.85 ~ 1.16)	1.01 (0.89 ~ 1.14)	0.88 (0.77 ~ 0.99)	0.96 (0.81 ~ 1.14)	0.90 (0.75 ~ 1.07)	0.93 (0.80 ~ 1.07)	0.89 (0.73 ~ 1.08)
Grade1(ref = Grade 4)	1.63 (1.14 ~ 2.33)**	1.47 (1.14 ~ 1.91)**	1.05 (0.82 ~ 1.34)	1.70 (1.10 ~ 2.62)*	1.73 (1.13 ~ 2.64)**	1.31 (0.97 ~ 1.77)	1.74 (1.08 ~ 2.81)*
Grade 2(ref = Grade 4)	1.56 (1.08 ~ 2.26)*	1.44 (1.10 ~ 1.87)**	1.14 (0.88 ~ 1.47)	1.79 (1.15 ~ 2.79)*	1.67 (1.08 ~ 2.59)*	1.28 (0.94 ~ 1.76)	1.68 (1.03 ~ 2.74)*
Grade 3(ref = Grade 4)	1.72 (1.19 ~ 2.50)**	1.54 (1.18 ~ 2.01)**	1.34 (1.04 ~ 1.73)*	2.09 (1.34 ~ 3.24)**	1.92 (1.24 ~ 2.97)**	1.59 (1.16 ~ 2.17)***	2.11 (1.29 ~ 3.43)**
Left-behind experiences (ref = No)	1.09 (0.94 ~ 1.26)	1.12 (1.00 ~ 1.26)*	1.21 (1.08 ~ 1.36)**	1.13 (0.96 ~ 1.34)	1.16 (0.98 ~ 1.37)	1.20 (1.05 ~ 1.37)**	1.18 (0.98 ~ 1.41)
City resident (ref = Village)	1.15 (0.98 ~ 1.35)	0.98 (0.86 ~ 1.10)	0.92 (0.82 ~ 1.05)	1.18 (0.96 ~ 1.41)	1.15 (0.96 ~ 1.37)	1.05 (0.91 ~ 1.21)	1.23 (1.01 ~ 1.50)*
Single child (ref = No)	0.90 (0.77 ~ 1.05)	0.95 (0.85 ~ 1.08)	0.88 (0.77 ~ 0.99)*	0.87 (0.73 ~ 1.03)	0.84 (0.71 ~ 1.00)	0.86 (0.75 ~ 0.99)*	0.82 (0.68 ~ 0.99)*
Family month income (ref = > 5000 Yuan)	1.20 (1.03 ~ 1.38)*	1.14 (1.02 ~ 1.28)*	1.09 (0.97 ~ 1.22)	1.28 (1.09 ~ 1.51)**	1.23 (1.06 ~ 1.47)*	1.18 (1.03 ~ 1.35)*	1.33 (1.12 ~ 1.60)**
Previous trauma experiences (ref = No)	0.86 (0.74 ~ 0.99)	1.13 (1.01 ~ 1.27)*	1.25 (1.12 ~ 1.40)***	0.94 (0.80 ~ 1.11)	1.00 (0.85 ~ 1.17)	1.29 (1.13 ~ 1.46)***	1.07 (0.90 ~ 1.27)
Pandemic exposure	1.10 (1.04 ~ 1.16)**	1.04 (0.99 ~ 1.08)	1.10 (1.05 ~ 1.15)***	1.06 (1.00 ~ 1.14)	1.12 (1.05 ~ 1.19)**	1.06 (1.00 ~ 1.11)	1.07 (1.00 ~ 1.15)
Rumination	1.17 (1.15 ~ 1.20)***	1.14 (1.13 ~ 1.17)***	1.22 (1.19 ~ 1.24)***	1.19 (1.16 ~ 1.22)***	1.20 (1.17 ~ 1.24)***	1.20 (1.18 ~ 1.23)***	1.21 (1.17 ~ 1.24)***
Self-disclosure	0.92 (0.91 ~ 0.93)***	0.92 (0.91 ~ 0.92)***	0.94 (0.93 ~ 0.95)***	0.92 (0.91 ~ 0.93)***	0.92 (0.91 ~ 0.93)***	0.92 (0.91 ~ 0.93)***	0.92 (0.91 ~ 0.93)***

Note: * p < 0.05, **p < 0.01,***p < 0.001; “a” represent that the reference group is non-PTSD, “b” represent that the reference group is non-depression, “c” represent that the reference group is non-anxiety, “d” represent that the reference group is non-comorbid PTSD and depression, “e” represent that the reference group is non-comorbid PTSD and anxiety, “f” represent that the reference group is non-comorbid depression and anxiety, “g” represent that the reference group is non-comorbid PTSD, depression, and anxiety.

## Discussion

This is a large cross-sectional investigation of college students’ PTSD, depression, and anxiety levels during the COVID-19 pandemic. We found that the prevalence of PTSD, depression, and anxiety was 15.5%, 32.2%, and 32.1%, respectively, which were lower than the findings of previous studies in college students [12, 13, 48]. One possible explanation is that previous studies carried out their investigations during the three months after the pandemic’s outbreak, whereas this study investigated college students about seven months following the pandemic’s outbreak. Therefore, the students in this study may have had more time to adapt to the pandemic than the college students in previous studies.

Moreover, we found that the prevalence of comorbid PTSD and depression, comorbid PTSD and anxiety, comorbid depression and anxiety, and comorbid PTSD, depression, and anxiety symptoms were 11.5%, 11.6%, 20.4%, and 9.4%, respectively. These findings indicated that there is higher prevalence of comorbidity between distinct psychiatric disorders during the COVID-19 pandemic, which is consistent with Karatzias ’s finding that many outcomes were comorbid in individuals [24]. Furthermore, this finding suggested that individuals who are experiencing traumatic stress due to the COVID-19 pandemic are likely to also be suffering from anxiety and depression [24].

Regarding for the predictors of these symptoms and their comorbidity, we did not find a significant gender effect, which is consistent with Qiu ’s study [48]. Similar results were also found in the analysis of the effect of rural or urban home environment on these symptoms. These results suggested that the psychological distresses due to the COVID-19 pandemic did not vary with gender or urban vs. rural areas, informing a large scope effect of this pandemic. Of course, further rationale for this finding needs to be discussed in future studies. We found that age may be correlated with these psychiatric disorders, as the college students who were in grade 4 were less likely to report PTSD, depression, anxiety, and their comorbid symptoms. These findings suggested that the oldest age categories were less likely to show COVID-19 related psychological distress, which is consistent with previous studies [24, 49]. This may be attributed to the possibility that older individuals have a relatively mature cognitive and emotional regulation capability and abundant life experiences. Thus, they are more likely to effectively handle the stressors caused by the COVID-19 pandemic.

Moreover, we also found that the students who were left behind and whose families had lower monthly incomes (e.g., lower than 5,000 RMB) were more likely to report more PTSD, anxiety, depression, and their comorbid symptoms. The left-behind status and lower family monthly incomes naturally indicated fewer available resources, but these resources may also have been further depleted by this pandemic. As a meta-analysis has highlighted, individuals with a higher socio-economic status may use better coping strategies due to greater social and economic resources, resulting in less impact from environmental disasters and ultimately reducing the prevalence of PTSD [50].

According to conservation of resources theory [51], this depletion and scarcity would add to the psychological distress experienced by these students, and getting more resources may relieve some of the negative effects of trauma on these individuals. The only children in this study fared a bit better psychologically, most likely because their parents were able to provide them with more resources. We found that students with previous traumatic experiences exhibited more symptoms of psychiatric disorders, which is consistent with previous studies related to trauma stress [52, 53]. This may be because previous trauma can be re-experienced at the physical and emotion levels, creating a sensitizing effect to new traumas which leads to higher rates of psychological distress [32].

In addition to these pre-disaster factors, we also assessed the role of within-disaster factors (e.g., pandemic exposure) and post-disaster factors (e.g., rumination and self-disclosure) in these three disorders as well as their comorbid symptoms. The results indicated that pandemic exposure was related to more PTSD, anxiety, and the comorbid symptoms of both. The COVID-19 pandemic has caused significant disruptions to people’s daily lives, including increased stress and anxiety levels due to concerns about the virus, social isolation, economic uncertainty, and the loss of loved ones [54]; College students who are exposed to the pandemic, whether through being infected with COVID-19, losing a loved one to the virus, or experiencing other pandemic-related stressors, may be at increased risk for developing PTSD and anxiety disorders [55]. The fear of contracting the virus, uncertainty about the future, and social isolation can also exacerbate existing symptoms of anxiety and PTSD and their comorbitity [56–59]. Additionally, we also found that rumination was positively correlated with these symptoms, but self-disclosure was negatively correlated. Rumination enhances negative thinking, impairs problem solving, and interferes with instrumental behaviors, and thus maintains and exacerbates psychological distress. symptomatology [48, 60]. In contrast, self-disclosure allows individuals to share their experiences with others, which strengthens social bonds and feelings of intimacy with others [61], increases the social cohesion of communities [61], and activates communal coping [62], all of which are mitigating factors for posttraumatic psychological distress [36].

Several limitations should be noted in this study. First, this study only assesses college students’ symptoms of psychiatric disorders including PTSD, depression, and anxiety about seven months following the COVID-19 outbreak. Thus, caution should be taken with the generalization of results to other samples in other time-points following this pandemic. Second, although the effects of pre- and post-disaster factors were examined, these are limited and more factors in pre- or post-disaster factors should be considered. Third, the cross-sectional design limits our understanding of the trajectory of college students’ PTSD, depression, and anxiety as well as their comorbid symptoms.

Despite these limitations, this study found a higher prevalence of comorbid PTSD, depression, and anxiety in college students during the COVID-19 pandemic, suggesting distinct psychological distresses may be exacerbated by comorbidity in individuals. Moreover, this study found that left-behind status, lower economic status, previous trauma experiences, exposure to the pandemic, and rumination were risk factors of psychological distress. Therefore, psychological services should give attention to comorbid symptoms as well as single symptoms following traumatic events, and targeted intervention should be directed toward left-behind students, students with lower economic status, previous trauma experiences, students with more exposure to pandemic, and those with higher rates of rumination. Because self-disclosure was a protective factor of these psychological distresses, helping students to learn to communicate their feelings to others may be an effective method of relieving psychological distress.

## Conclusion

This study sheds light on PTSD, depression, anxiety and their comorbidities during the COVID-19 pandemic. Our results showed that the pandemic has already had a great psychological impact on the Chinese college students. Crucially, psychological distresses may be exacerbated by comorbidity in individuals. Moreover, it has been highlighted that left-behind status, lower economic status, previous trauma experiences, exposure to the pandemic, and rumination were risk factors of psychological distress. But self-disclosure was a protective factor for these disorders. Thus, mental health professionals need to recognize individuals who are at a higher risk of comorbid symptoms rather than single symptom of single disorders, so as to prevent a possible rise of high post-traumatic stress for future infectious disease outbreaks.

## Data Availability

The datasets used and/or analysed during the current study available from the corresponding author on reasonable request.
